# Age-related differences in choroid plexus structural integrity are associated with changes in cognition

**DOI:** 10.1186/s12987-025-00749-3

**Published:** 2025-12-18

**Authors:** Zhaoyuan Gong, Angelique de Rouen, Nathan Zhang, Joseph S.R. Alisch, Murat Bilgel, Yang An, Jonghyun Bae, Noam Y. Fox, Alexander Y. Guo, Susan M. Resnick, Caio H. Mazucanti, Samuel Klistorner, Alexander Klistorner, Josephine M. Egan, Mustapha Bouhrara

**Affiliations:** 1 Laboratory of Clinical Investigation, National Institute on Aging, National Institutes of Health, Baltimore, MD 21224 USA; 2 Laboratory of Behavioral Neuroscience, National Institute on Aging, National Institutes of Health, Baltimore, MD 21224 USA; 3 Save Sight Institute, Sydney Medical School, University of Sydney, Sydney, New South Wales Australia

**Keywords:** Choroid plexus, Microstructure, MRI, Biomarker, Cognition, Longitudinal

## Abstract

**Background:**

The choroid plexus (CP) plays a critical role in maintaining central nervous system (CNS) homeostasis, producing cerebrospinal fluid, and regulating the entry of specific substances into the CNS from blood. CP dysfunction has been implicated in various neurological and psychiatric disorders, including Alzheimer’s disease, Parkinson’s disease, and multiple sclerosis.

**Methods:**

This study investigates the relationship between CP structural integrity and cognitive decline in normative aging, using structural and advanced magnetic resonance imaging techniques, including CP volume, diffusion tensor imaging indices (mean diffusivity, MD, and fractional anisotropy, FA) and relaxometry metrics (longitudinal, T_1_, and transverse, T_2_, relaxation times).

**Results:**

Our results show that lower CP microstructural integrity, as reflected by higher T_1_, T_2_, and MD values, or lower FA values, is associated with lower cognitive performance in processing speed and fluency. Notably, CP microstructural measures demonstrated greater sensitivity to cognitive decline than macrostructural measures, i.e. CP volume. Longitudinal analysis revealed that individuals with lower CP structural integrity exhibit steeper cognitive decline over time. Furthermore, structural equation modeling revealed that a latent construct representing CP integrity predicts faster overall cognitive decline, with an effect size comparable to that of age.

**Conclusions:**

These findings highlight the importance of CP integrity in maintaining cognitive health and suggest that a holistic approach to assessing CP integrity could serve as a sensitive biomarker for early detection of cognitive decline. Further research is needed to elucidate the mechanisms underlying the relationship between CP structural integrity and clinical decline and to explore the potential therapeutic implications of targeting CP function to prevent or treat age-related cognitive deficits.

**Supplementary information:**

The online version contains supplementary material available at 10.1186/s12987-025-00749-3.

## Background

The choroid plexus (CP), a highly vascularized structure within the brain ventricles, is responsible for producing cerebrospinal fluid (CSF) by specialized epithelial cells. This process maintains the optimal composition and volume of CSF, providing essential cushioning and protection for the central nervous system (CNS) [1, 2]. It also acts as a selective barrier between the blood and CSF, regulating the entry of substances into the CNS. Additionally, the CP structure facilitates bulk removal of waste products through the glymphatic system and plays pivotal roles in regulating chemical balance, ionic composition, immunosurveillance, and influencing circadian rhythmicity [3–5]. Dysregulation of these CNS processes can cause cerebral tissue degeneration and neuroinflammatory responses, leading to disrupted neural signaling as well as concomitant cognitive impairments and motor dysfunction [6–8]. Indeed, CP integrity is believed to play a significant role in cognition, as increasing research indicates that an enlarged CP is often associated with cognitive impairment in a myriad of conditions including mild cognitive impairment [9], Alzheimer’s disease (AD) [10, 11], Parkinson’s disease (PD) [12, 13], and multiple sclerosis (MS) [14, 15]. A larger CP may indicate a disruption in the brain’s clearance system, potentially contributing to clinical deficits [9, 16]. Therefore, measuring macrostructural and microstructural changes of CP through advanced neuroimaging techniques, notably magnetic resonance imaging (MRI), could potentially develop biomarkers for early detection of cognitive and functional decline. However, there is a paucity of research investigating the relationship between CP structural health and clinical impairment in normative aging, particularly from a longitudinal perspective. Importantly, microstructural changes in the CP are likely to precede macrostructural changes (i.e., volume changes), making it crucial to investigate microstructural alterations in relation to cognitive and functional changes. Elucidating the role of the CP structural integrity in cognition could lead to the development of novel therapeutic strategies aimed at improving its function, offering promising avenues for early intervention and prevention of cognitive decline.

Established quantitative MRI (qMRI) techniques, including diffusion tensor imaging (DTI) metrics (mean diffusivity, MD, and fractional anisotropy, FA) and relaxometry indices (longitudinal relaxation time, T_1_, and transverse relaxation time, T_2_), enable assessment of differences in CP microstructural integrity with aging, obesity, and markers of neuroinflammation and AD pathology [17–21]. DTI is sensitive to the underlying microarchitectural status of the CNS tissue and the degree and direction of water molecule mobility [22], and has been extensively used to study brain maturation and degeneration [23]. Reduced FA is associated with structural degeneration, whereas an increase in MD reflects damage to cerebral tissue microstructural integrity with increased water mobility. Similarly, T_1_ and T_2_ both depend on macromolecular tissue composition, biochemistry and water mobility [24]. Changes in T_1_ or T_2_ are directly associated with cerebral microstructural tissue changes, where higher values indicate advanced cerebral tissue deterioration [24]. Notably, these associations between qMRI metrics and cerebral microstructural changes during aging are also observed in CP [18], reinforcing the potential of qMRI for monitoring CP microstructural alterations. While studies using structural MRI have revealed that greater CP volume is inversely correlated with cognitive performance including in the AD continuum [9, 25], studies investigating microstructural differences in CP and cognition, as probed using DTI or relaxometry metrics, are lacking, including in normative aging. Yet, such investigations would further our understanding of the complex relationships between CP microstructure, function, and cognitive health [21].

This study aims to investigate the relationship between CP structural integrity, cognitive function and aging, leveraging both structural MRI and qMRI techniques. Specifically, we examined CP microstructural differences associated with normative aging and longitudinal cognitive decline, and assessed the utility of diffusion and relaxometry metrics as biomarkers for early cognitive decline detection. This work advances our understanding of the complex interplay between CP structure and cognitive health.

## Methods

### Participants

The MRI protocol received approval from the MedStar Research Institute and the National Institutes of Health (NIH) Intramural Ethics Committees. All examinations adhered to the standards set by the NIH Institutional Review Board. Study participants were recruited from the Baltimore Longitudinal Study of Aging (BLSA) and the Genetic and Epigenetic Signatures of Translational Aging Laboratory Testing (GESTALT) study [26, 27]. Details regarding the study population, experimental design, and measurement protocols of BLSA and GESTALT have been described in previous publications [26, 27]. The BLSA is a longitudinal cohort study initiated in 1958, conducted and funded by the National Institute on Aging (NIA) Intramural Research Program (IRP). The BLSA enrolls healthy, community-dwelling adults without major chronic conditions or functional impairments. Similarly, the GESTALT study, launched in 2015, is also conducted and funded by the NIA IRP, with virtually identical inclusion and exclusion criteria. Participants in both studies underwent evaluations at the NIAs clinical research unit, with exclusions based on metallic implants, neurological or medical disorders, and cognitive impairment, as assessed through a comprehensive battery of cognitive tests [28]. Nine participants were excluded due to cognitive impairment related to mild cognitive impairment or dementia.

### Magnetic resonance imaging (MRI)

MRI scans were performed on a 3T whole-body Philips MRI system (Achieva, Philips Healthcare, Best, The Netherlands), utilizing the internal quadrature body coil for transmission and an eight-channel phased-array head coil for signal reception. For each participant, the imaging protocol for longitudinal and transverse relaxation times (T_1_, T_2_) and DTI metrics (FA and MD) consisted of:

#### T_1_ and T_2_ mapping [29–32]

3D spoiled gradient recalled echo (SPGR) images were acquired with flip angles (FAs) of [2 4 6 8 10 12 14 16 18 20]°, echo time (TE) of 1.37 ms, repetition time (TR) of 5 ms, and acquisition time of ~ 5 min. Additionally, 3D balanced steady-state free precession (bSSFP) images were acquired with FAs of [2 4 7 11 16 24 32 40 50 60]°, TE of 2.8 ms, TR of 5.8 ms, and acquisition time of ~ 6 min. To account for off-resonance effects, bSSFP images were acquired with radiofrequency excitation pulse phase increments of 0 or π [33]. All SPGR and bSSFP images had an acquisition matrix of 150 × 130 × 94, voxel size of 1.6 mm × 1.6 mm × 1.6 mm. The double-angle method (DAM) was used to correct for excitation radiofrequency inhomogeneity [34]. For this, two fast spin-echo images were acquired with FAs of 45° and 90°, TE of 102 ms, TR of 3000 ms, acquisition voxel size of 2.6 mm × 2.6 mm × 4 mm, and acquisition time of ~ 4 min. All images were acquired with field of view (FoV) of 240 mm × 208 mm × 150 mm.

#### FA and MD mapping [23, 35]

DTI protocol consisted of diffusion-weighted images (DWI) acquired with single-shot Echo Planar Imaging (EPI), TR of 10,000 ms, TE of 70 ms, two b-values of 0 and 700 s/mm^2^, with the latter encoded in 32 directions. The acquisition matrix was 120 × 104 × 75, with voxel size of 2 mm × 2 mm × 2 mm, with FoV of 240 mm × 208 mm × 150 mm. DTI datasets were acquired in accordance with the standardized BLSA/GESTALT diffusion MRI protocol. The moderate diffusion weighting (b = 700 s/mm^2^) was chosen to maintain a high signal-to-noise ratio and reduce susceptibility- and motion-related artifacts, particularly in small and CSF-proximal regions such as the choroid plexus. This b-value has been widely adopted in large-scale aging studies, ensuring data comparability and reliable tensor estimation.

### MRI processing

MRI processing and analysis details can be found in Reference [18]. In brief, scalp and nonparenchymal regions were eliminated using FSL. CP volume was calculated using FreeSurfer for each participant. CP masks were thoroughly examined and corrected manually when needed. T_1_ and T_2_ mapping as well as FA and MD mapping, were performed using FSL and in-house MATLAB scripts. Mean T_1_, T_2_, FA, and MD values were extracted from the CP region of interest (ROI) using the CP masks from FreeSurfer. While examples of CP ROI location has been reported in our previous work [18], qMRI maps at different ages are presented in supplementary Figure S1 to illustrate the age-related differences in CP qMRI metrics.

### Cognitive assessment

Cognitive domain scores were obtained for memory (California Verbal Learning Test [36] immediate and long-delay free recall), attention (Trail Making Test [37] Part A and Digit Span [38] Forward), executive function (Trail Making Test Part B and Digit Span Backward), verbal fluency (Category [39] and Letter Fluency [40]), and processing speed (Trail Making Test [37] Part A and Digit Symbol Substitution Test). To obtain domain scores, each test score was first converted to a z-score using the baseline mean and standard deviation, and these z-scores were averaged within each cognitive domain. Before computing the z-scores for Trail Making Test Parts A and B, the individual cognitive test scores (time to completion, in seconds) were log-transformed and negated so that higher z-scores indicated shorter time to completion.

### Statistical analysis

We conducted three statistical analyses to assess the relationship between CP and cognition. All reported p-values in the main manuscript are adjusted for multiple comparisons across all regressions using the Benjamini & Hochberg procedure. Analyses were programmed in R 4.4.2, with lme4, lavaan, and tidyverse packages.

#### CP structural integrity vs. cognition

To assess the cross-sectional relationship between CP structural integrity and cognition, we paired the MRI scan with the closest cognition scores in time to create cross-sectional datasets. We then performed multiple linear regressions to evaluate the association between each MRI metric and cognitive domain, specified as:

1 $$\begin{aligned}{\rm{Cognition}} \sim \,&{{\rm{\beta }}_{\rm{0}}}\,{\rm{ + }}\,{{\rm{\beta }}_{{\rm{MRI}}}}\,{\rm{ \times }}\,{\rm{MRI}}\,{\rm{ + }}\,{{\rm{\beta }}_{{\rm{Age}}}}\,{\rm{ \times }}\,{\rm{Age}}\,{\rm{ + }}\,{{\rm{\beta }}_{{\rm{Sex}}}}\,\\&{\rm{ \times }}\,{\rm{Sex}}\,{\rm{ + }}\;{{\rm{\beta }}_{{\rm{EDU}}}}\,{\rm{ \times }}\,{\rm{EDU}}\,{\rm{ + }}\,{{\rm{\beta }}_{{\rm{Race}}}}\,{\rm{ \times }}\,{\rm{Race}}\,{\rm{ + }} \in {\rm{.}}\end{aligned}$$

where Cognition represents one of five cognitive domains: processing speed (PS), memory (MEM), verbal fluency (FLU), executive function (EF), or attention (ATT). MRI metrics include CP volume (Vol), longitudinal relaxation time (T_1_), transverse relaxation time (T_2_), fractional anisotropy (FA), and mean diffusivity (MD). We observed collinearity between MRI metrics and Age, which weakened the significance of expected age-related cognitive declines. To address this, we regressed MRI metrics on covariates and replaced the original MRI values with their residuals. Covariates included Age (at the time of MRI), Sex (coded with male as the reference group), EDU (years of education), and Race (categorized as White, Black, or Other, with White as the reference group). All continuous variables were standardized for analysis.

####  CP structural integrity vs. changes in cognition

To investigate the relationship between CP integrity and longitudinal cognitive changes, we constructed longitudinal datasets by pairing repeated cognitive assessments with each participant’s single MRI scan. The longitudinal dataset comprised 320 observations from 116 unique participants. We employed linear mixed-effects models:

2 $$\begin{aligned}{\rm{Cognition}}_{ij} \sim\,& {{\rm{\beta }}_{\rm{0}}}\,{\rm{ + }}\,{{\rm{\beta }}_{{\rm{Time}}}}\,{\rm{ \times }}\,{\rm{Tim}}{{\rm{e}}_{ij}}\,{\rm{ + }}\,{{\rm{\beta }}_{{\rm{MRI}}}}\,{\rm{ \times }}\,{\rm{MR}}{{\rm{I}}_i}\,\\&{\rm{ + }}\,{{\rm{\beta }}_{{\rm{Time \times MRI}}}}\,{\rm{ \times }}\,{\rm{Tim}}{{\rm{e}}_{ij}}\,{\rm{ \times }}\,{\rm{MR}}{{\rm{I}}_i}\,{\rm{ + }}\;{{\rm{\beta }}_{{\rm{Age}}}}\,\\&{\rm{ \times }}\,{\rm{Ag}}{{\rm{e}}_i}\,{\rm{ + }}\,{{\rm{\beta }}_{{\rm{Sex}}}}\,{\rm{ \times }}\,{\rm{Se}}{{\rm{x}}_i}\,{\rm{ + }}{{\rm{\beta }}_{{\rm{EDU}}}}\,\\&{\rm{ \times }}\,{\rm{ED}}{{\rm{U}}_i}\,{\rm{ + }}\,{{\rm{\beta }}_{{\rm{Race}}}}\,{\rm{ \times }}\,{\rm{Rac}}{{\rm{e}}_i}\,{\rm{ + }}\,{ \in _{ij}}\,{\rm{ + }}\,{{\rm{b}}_i}{\rm{,}}\end{aligned}$$

where Cognition_*ij*_ represents the cognitive score for subject *i* at time point *j* in PS, MEM, FLU, EF, or ATT. Time_*ij*_ is the Time-to-MRI calculated as the time difference between the time point *j* and the time at the MRI scan for subject *i*. MRI_*i*_ denotes the MRI metric for subject *i* at the time of the MRI scan. The interaction term Time_*ij*_×MRI_*i*_ tests whether MRI metrics modulate the rate of cognitive change over time. *b*_*i*_ is the random intercept for subject *i*, and ϵ_*ij*_ is the residual error for subject *i* at time point *j*. Age_*i*_, Sex_*i*_, EDU_*i*_, and Race_*i*_ were included as covariates, as previously defined.

#### Overall CP integrity and overall cognitive function

We employed structural equation modeling (SEM) to investigate the relationships between latent constructs representing CP integrity and cognitive decline. SEM is a comprehensive statistical approach that facilitates the analysis of complex relationships involving both measured variables and unobserved constructs (latent variables). In this study, we opted to construct two comprehensive latent variables: CP Integrity (CPI) and Cognitive Function (CogFun). The CPI latent variable represents the unobservable overall structural integrity of the CP, inferred collectively from macrostructural and microstructural MRI measurements. In contrast, CogFun represents the unobservable overall rate of cognitive function changes, derived from all rates of change in the evaluated cognitive domains. By modeling these latent variables, we aimed to holistically assess the impact of CPI on overall cognitive function changes. The measurement model for CPI (exogenous latent variable) is defined as:

3 $$\begin{aligned}{\rm{CPI}}\;{\rm{ = }}\;& \sim {{\rm{\lambda }}_{{{\rm{T}}_{\rm{1}}}}}\;{\rm{ \times }}\;{{\rm{T}}_{\rm{1}}}\;{\rm{ + }}\;{{\rm{\lambda }}_{{{\rm{T}}_{\rm{2}}}}}\;{\rm{ \times }}\;{{\rm{T}}_{\rm{2}}}\;{\rm{ + }}\;{{\rm{\lambda }}_{{\rm{FA}}}}\;\\&{\rm{ \times }}\;{\rm{FA}}\;{\rm{ + }}\;{{\rm{\lambda }}_{{\rm{MD}}}}\; \times \;{\rm{MD}}\;{\rm{ + }}\;{{\rm{\lambda }}_{{\rm{Vol}}}}\; \times \;{\rm{Vol}}\;{\rm{ + }}\; \in, \end{aligned}$$

where λs represent factor loadings, and ϵ represents residual variance. In this study, λs were estimated by maximum likelihood. Loadings quantify the relation between each observed indicator and their latent construct; larger |λ| denotes a stronger reflection of the latent variable. The latent scale was identified by fixing a marker loading to 1, and we reported fully standardized estimates (Std.all) for interpretability.

Similarly, the measurement model for CogFun (endogenous latent variable) is defined as:

4 $$\begin{aligned}{\rm{CogFun}}\,{\rm{ = }}\,&\sim\,{{\rm{\lambda }}_{{\rm{MEM}}}}\,{\rm{ \times }}\,{{\rm{R}}_{{\rm{MEM}}}}\,{\rm{ + }}\,{{\rm{\lambda }}_{{\rm{ATT}}}}\,{\rm{ \times }}\,{{\rm{R}}_{{\rm{ATT}}}}\,{\rm{ + }}\,{{\rm{\lambda }}_{{\rm{EF}}}}\,\\&{\rm{ \times }}\,{{\rm{R}}_{{\rm{EF}}}}\,{\rm{ + }}\,{{\rm{\lambda }}_{{\rm{FLU}}}}\,{\rm{ \times }}\,{{\rm{R}}_{{\rm{FLU}}}}\,{\rm{ + }}\,{{\rm{\lambda }}_{{\rm{PS}}}}\,{\rm{ \times }}\,{{\rm{R}}_{{\rm{PS}}}}\,{\rm{ + }}\, \in ,\end{aligned}$$

where R_MEM_, R_ATT_, R_EF_, R_FLU_, R_PS_ are rates of change in memory, attention, executive function, fluency, and processing speed, respectively. We modeled the structural relationship between CP Integrity and cognitive function as:

5 $$\begin{aligned}{\rm{CogFun}}\, &\sim \,{{\rm{\beta }}_{{\rm{CPI}}}}\,{\rm{ \times }}\,{\rm{CPI}}\,{\rm{ + }}\,{{\rm{\beta }}_{{\rm{Age}}}}\,{\rm{ \times }}\,{\rm{Age}}\,{\rm{ + }}\,{{\rm{\beta }}_{{\rm{Sex}}}}\,\\&{\rm{ \times }}\,{\rm{Sex}}\,{\rm{ + }}\,{{\rm{\beta }}_{{\rm{EDU}}}}\,{\rm{ \times }}\,{\rm{EDU}}\,{\rm{ + }}\,{{\rm{\beta }}_{{\rm{Race}}}}\,{\rm{ \times }}\,{\rm{Race}}\,{\rm{ + }}\, \in .\end{aligned}$$

All results are presented in standardized form, with variables scaled to have a mean of zero and a standard deviation of one. This scaling allows parameter estimates (e.g., factor loadings, regression coefficients, and variances) to be interpreted as standardized effects.

## Results

Table 1 provides an overview of the study demographics. A total of 116 subjects were included. In the cross-sectional cohort, the median time between cognitive testing and MRI scanning was 0 years (mean: −0.17 years; SD: 0.6 years). In the longitudinal cohort, participants underwent an average of 2.76 cognitive assessments (SD: 2.74), with a median follow-up duration of 2.2 years. The average age at the time of MRI was 57.2 years, and 44.8% of the participants were female. The cohort’s mean education level was 16.2 years (SD: 2.8 years). The cohort comprised 70.7% of white participants, indicating an imbalance in racial representation. Of the 116 participants, 76 had at least one follow-up cognitive assessment (mean interval = 4.23 ± 6.03 years, range = 0–23.5 years). For descriptive purposes, baseline and last available cognitive scores were used to compute change over time. Mean changes in scores for each domain are presented in Table 1. Supplementary Figures S2-S4 provide additional context for interpreting the main analyses. Specifically, Supplementary Figure S2 illustrates the correlations between cognitive scores, age, CP MRI metrics, and covariates in the cross-sectional dataset, offering insight into the interrelationships among key variables. Supplementary Figure S3 depicts cognitive score trajectories as a function of age in the longitudinal dataset with the time of MRI scan denoted, visually demonstrating patterns of decreasing cognition with age and supporting the interpretation of temporal effects observed in the main results. Supplementary Figure S4 presents the pairwise correlations among cognitive measures, CP MRI metrics, and the quadratic age term, highlighting nonlinear age-related associations within the cross-sectional data.

**Table 1:** Participant demographics for cross-sectional and longitudinal analysis

Characteristic	Cross-sectional analysis (*N* = 116)	Longitudinal analysis (*N* = 116, *N*_cog_ = 320)
Age at MRI (years), mean (SD)	57.2 (20.9)	
Sex Female, n (%)	52 (44.8%)	
Education, mean (SD)	16.2 (2.8)	
Race, Black	22 (19%)	
White	82 (70.7%)	
API and Other	12 (10.3%)	
Number of cognitive assessments, mean (SD)	N/A	2.76 (2.74)
Duration of cognitive assessments (years), median (mean, SD, min, max)	N/A	2.2 (4.23, 6.03, 0, 23.5)
Cognition z-score changes, mean (SD)		
MEM	N/A	−0.083 (0.52)
FLU		−0.061 (0.36)
EF		−0.054 (0.47)
ATT		−0.054 (0.41)
PS		−0.187 (0.54)

N, number of participants. N_cog_, number of cognitive assessments. MRI, magnetic resonance imaging. API, Asian and Pacific Islander. SD, standard deviation. MEM, memory. FLU, verbal fluency. EF, executive function. ATT, attention. PS, processing speed

**Fig. 1:**
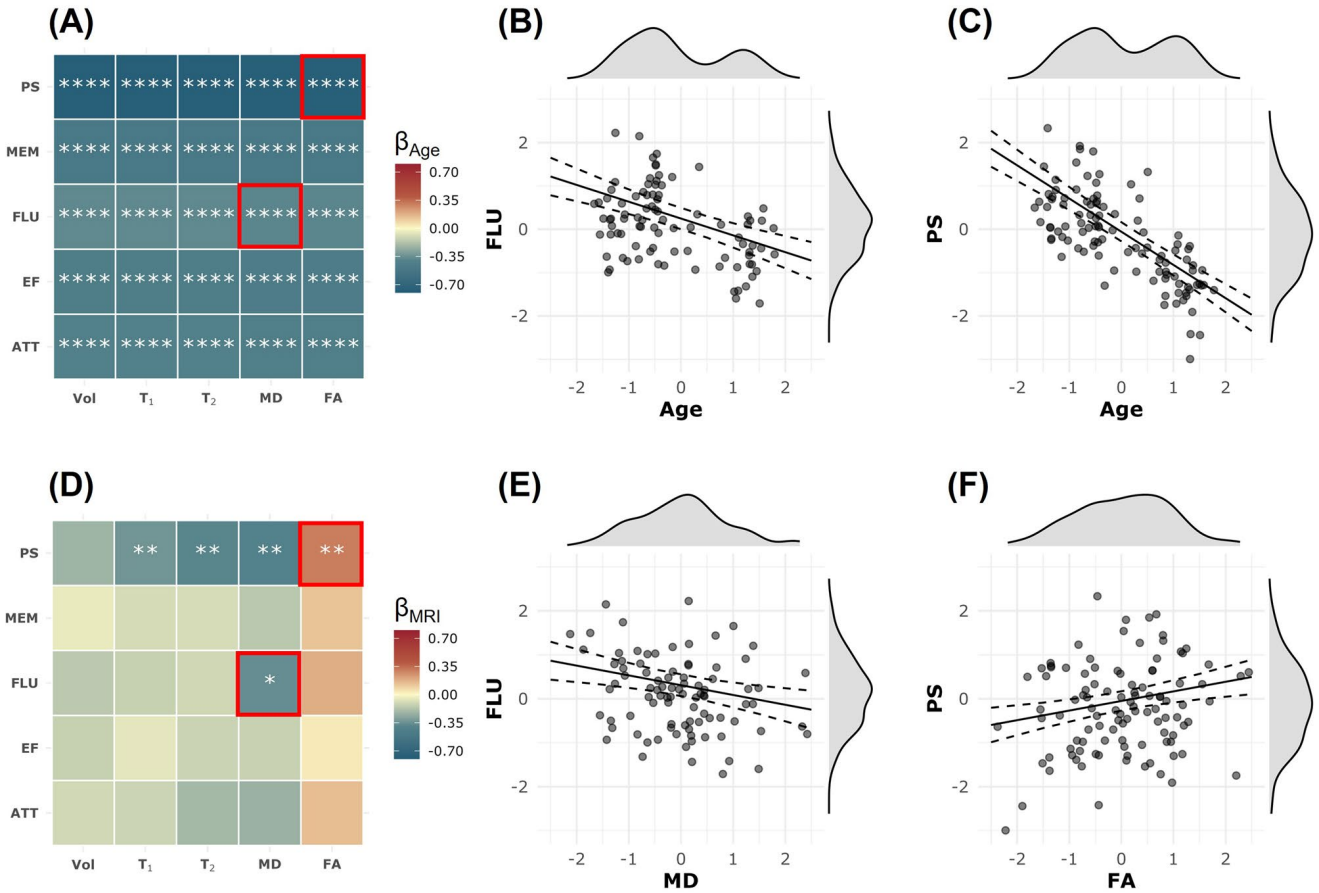
Pairwise multiple linear regression was conducted for each MRI metric and each cognitive domain. The explicit regression equation is: ${\rm{Cognition}}\; \sim \;{{\rm{\beta }}_{\rm{0}}}\;{\rm{ + }}\;{{\rm{\beta }}_{{\rm{MRI}}}}\;{\rm{ \times }}\;{\rm{MRI}}\;{\rm{ + }}\;{{\rm{\beta }}_{{\rm{Age}}}}\;{\rm{ \times }}\;{\rm{Age}}\;{\rm{ + }}\;{{\rm{\beta }}_{{\rm{Sex}}}}\;{\rm{ \times }}\;{\rm{Sex}}\;{\rm{ + }}\;{{\rm{\beta }}_{{\rm{EDU}}}}\;{\rm{ \times }}\;{\rm{EDU}}\;{\rm{ + }}\;{{\rm{\beta }}_{{\rm{Race}}}}\;{\rm{ \times }}\;{\rm{Race}}\;{\rm{ + }}\; \in$, where cognition includes processing speed (PS), memory (mem), verbal fluency (FLU), executive function (EF), and attention (ATT). MRI metrics include the residuals of CP volume (vol), T_1_ relaxation time (T_1_), T_2_ relaxation time (T_2_), fractional anisotropy (FA), and mean diffusivity (MD) after regressing with other covariates. All continuous variables are standardized. **Panels A and D** show the results for the regression coefficients of age and MRI metrics, respectively. The estimated β coefficients are color-coded and statistically significant results are marked with asterisks, where * indicates *p* < 0.05, ** indicates *p* < 0.01, *** indicates *p* < 0.001, and **** indicates *p* < 0.0001. All p-values are corrected for multiple comparisons using the Benjamini–Hochberg (BH) procedure. **Panels B and E** present example results for the regression where the MRI metric is MD and the cognitive domain is FLU. There is a significant negative association between FLU and age (Panel B) and a significant negative association between MD and FLU (Panel E). High MD values indicate reduced microstructural integrity, which is associated with lower FLU. **Panels C and F** show example results for the regression where the MRI metric is FA and the cognitive domain is PS. In addition to a significant negative association between PS and age (Panel C), there is a significant positive association between FA and PS (Panel F). High FA values indicate greater microstructural integrity, which is associated with higher PS scores

Figure 1 shows the results of the multiple linear regression analysis (Eq. 1) examining the cross-sectional relationship between each MRI metric (i.e., CP volume, T_1_, T_2_, MD, and FA) and each cognitive domain (i.e., PS, MEM, FLU, EF, and ATT). As expected, age was negatively associated with cognition in all examined regressions. These associations were all statistically significant. Examples of these associations are shown in Fig. 1B and C. Importantly, there was a trend for an association between CP changes and cognitive tests. Specifically, CP volume, T_1_, T_2_, and MD exhibited negative associations with cognition, while FA exhibited positive association with cognition, indicating that worse macrostructural CP integrity, represented by higher CP volume, and worse microstructural CP integrity, as measured by higher T_1_, T_2_, MD values and lower FA, are associated with worse cognition (Fig. 1D). However, these associations were statistically significant only for MD vs. FLU and PS as well as T_1_, T_2_, and FA vs. PS (Fig. 1D). Representative plots of these associations are shown in Fig. 1E and F. In addition, sex differences were significant, with males scoring higher in EF and ATT but lower in MEM. Higher education was significantly associated with better performance in MEM, FLU, EF, and ATT. White participants showed significantly better performance in ATT, EF, and PS. We note that the main purpose of this study is not to investigate the covariates, and the results may not be generalizable due to confounding factors. Complete analysis results are provided in the supplementary materials.

**Fig. 2:**
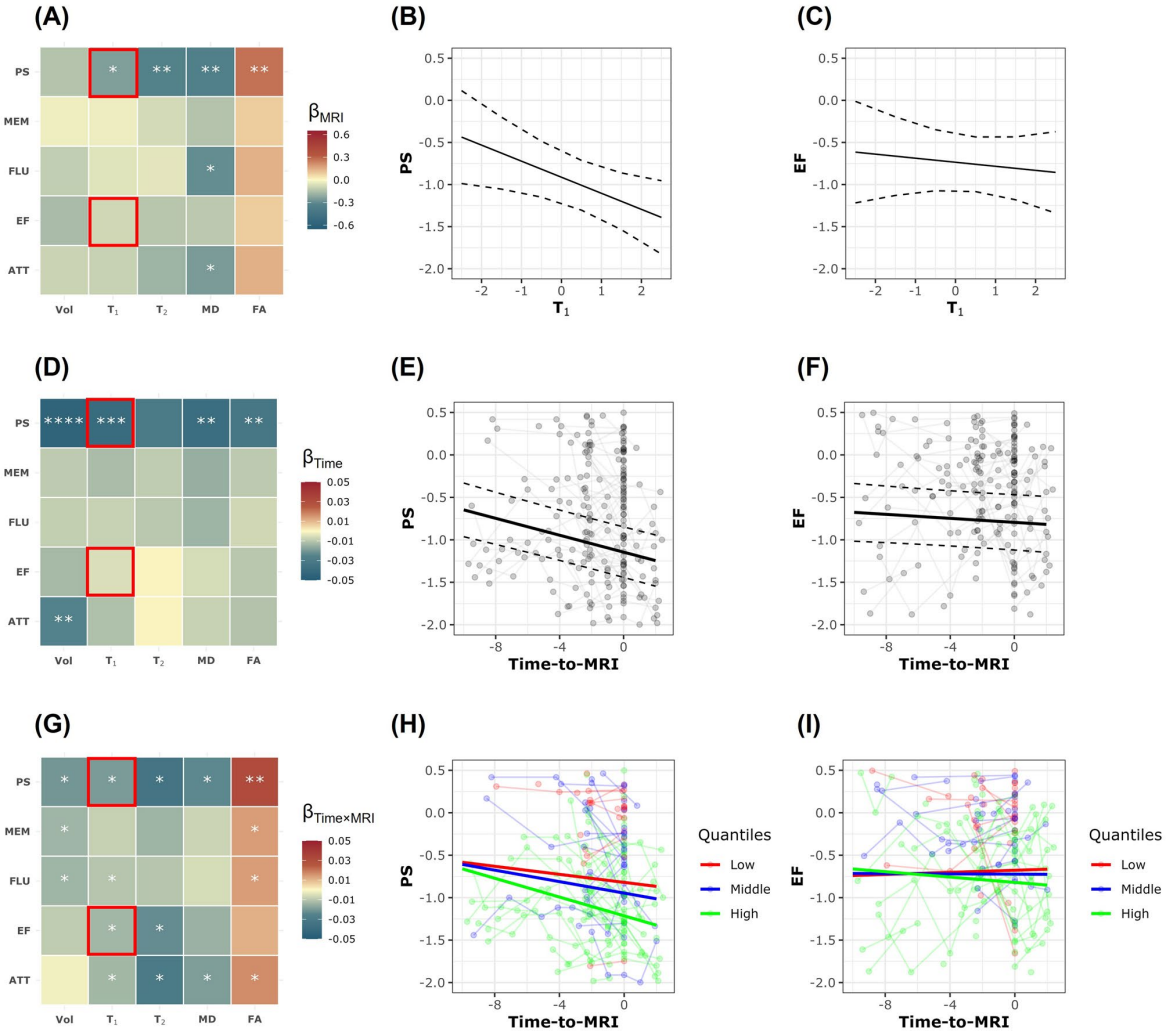
Pairwise linear mixed-effects (LME) models were conducted for each MRI metric and each cognitive domain. The explicit regression equation is: ${\rm{Cognitio}}{{\rm{n}}_{{\rm{ij}}}}\,{\rm{ = }}\,{{\rm{\beta }}_{\rm{0}}}\,{\rm{ + }}\,{{\rm{\beta }}_{{\rm{Time}}}}\,{\rm{ \times }}\,{\rm{Time}}{}_{{\rm{ij}}}\,{\rm{ + }}\,{{\rm{\beta }}_{{\rm{MRI}}}}\,{\rm{ \times }}\,{\rm{MR}}{{\rm{I}}_{\rm{i}}}\,{\rm{ + }}\,{{\rm{\beta }}_{{\rm{Time}}\,{\rm{ \times }}\,{\rm{MRI}}}}\,{\rm{ \times }}\,{\rm{Tim}}{{\rm{e}}_{{\rm{ij}}}}\,{\rm{ \times }}\,{\rm{MR}}{{\rm{I}}_{\rm{i}}}\,{\rm{ + }}\,{{\rm{\beta }}_{{\rm{Age}}}}\,{\rm{ \times }}\,{\rm{Ag}}{{\rm{e}}_{\rm{i}}}\,{\rm{ + }}\,{{\rm{\beta }}_{{\rm{Sex}}}}\,{\rm{ \times }}\,{\rm{Sex}}{}_{\rm{i}}\,{\rm{ + }}\,{{\rm{\beta }}_{{\rm{EDU}}}}\,{\rm{ \times }}\,{\rm{ED}}{{\rm{U}}_{\rm{i}}}\,{\rm{ + }}\,{{\rm{\beta }}_{{\rm{Race}}}}\,{\rm{ \times }}\,{\rm{Rac}}{{\rm{e}}_{\rm{i}}}\,{\rm{ + }}\,{ \in _{{\rm{ij}}}}\,{\rm{ + }}\,{{\rm{b}}_{\rm{i}}}$, where Cognition_ij_ includes longitudinal measurements of processing speed (PS), memory (mem), verbal fluency (flu), executive function (EF), and attention (ATT) for subject i at timepoint j. Time_ij_ represents the time to the single MRI scan of subject i at timepoint j. MRI metrics (MRI_i_) include CP volume (Vol), T_1_ relaxation time (T_1_), T_2_ relaxation time (T_2_), fractional anisotropy (FA), and mean diffusivity (MD) for subject i at the time of MRI scan. All continuous variables are standardized except for Time_ij_. **Panels A, D and G** show the results for the regression coefficients of MRI metrics, time and time × MRI interaction, respectively. The estimated β coefficients are color-coded and statistically significant results are marked with asterisks, where * indicates *p* < 0.05, ** indicates *p* < 0.01, *** indicates *p* < 0.001, and **** indicates *p* < 0.0001. All p-values are corrected for multiple comparisons using the Benjamini–Hochberg (BH) procedure. **Panels B, E, and H** present example results for the LME model where the MRI metric is T_1_ and the cognitive domain is PS. A significant negative association between the fixed effect of T_1_ and PS is observed (Panel B). This indicates that individuals with higher T_1_ values tend to have consistently lower cognitive scores over time. PS also shows a significant linear decline over time (Panel E). Most importantly, the significant negative interaction term indicates that individuals with higher T_1_ values experience faster declines in PS over time compared to those with lower T_1_ values (green line vs. red line in Panel H). **Panels C, F, and I** present example results for the LME model where the MRI metric is T_1_ and the cognitive domain is EF. No significant association is found between the fixed effect of T_1_ and EF (Panel C). EF also does not show a significant general decline over time (Panel F). However, a significant negative interaction term is observed, indicating that individuals with higher T_1_ values experience faster declines in EF over time compared to those with lower T_1_ values (green line vs. red line in Panel I). Increased T_1_ relaxation times could reflect microstructural damanges, which could impair cognitive processes and may exacerbate cognitive decline over time observed here

Figure 2 shows the results of the linear mixed-effects analysis (Eq. 2) examining the relationship between each MRI metric (i.e., CP volume, T_1_, T_2_, MD, and FA) and longitudinal changes in each cognitive domain (i.e., PS, MEM, FLU, EF, and ATT). Results are shown for the main parameters of interest relevant to this investigation, namely, MRI fixed effect, Time-to-MRI, and Time × MRI interaction. MRI metrics of CP volume, T_1_, T_2_, and MD exhibited negative associations with cognition, while FA exhibited a positive association with cognition, indicating that worse CP structural integrity is associated with lower cognitive performance. These associations reached statistical significance for MD vs. PS, FLU and ATT as well as T_1_, T_2_ and FA vs. PS (Fig. 2A). Examples of plots of these associations are shown in Fig. 2B and C. Further, as expected, Time-to-MRI exhibited overall negative association with cognition, reaching significance in various pairwise MRI metrics vs. cognitive domains as indicated in Fig. 2D–F. Importantly, the association between Time × MRI interaction and cognition exhibited negative associations for Vol, T_1_, T_2_ and MD and positive associations for FA (Fig. 2G). These associations were statistically significant for several MRI metrics vs. cognitive domains (Fig. 2G). These analyses indicate that lower CP structural integrity, as reflected by higher CP volume, T_1_, T_2_ and MD values or lower FA values, is associated with steeper longitudinal decline in cognition. Figure 2 H and I show the example of higher T_1_ value, corresponding to the value at the 75^th^ percentile marked with the green lines, is associated with steeper decline in PS and EF. Sex differences were significant, with males scoring higher in ATT but lower in MEM. Higher education was significantly associated with better performance in all cognitive domains, while White participants showed significantly better performance in ATT, EF, and PS. Same caution should be exercised for generalizing the results for covariates. Complete analysis results are provided in the supplementary materials.

**Fig. 3:**
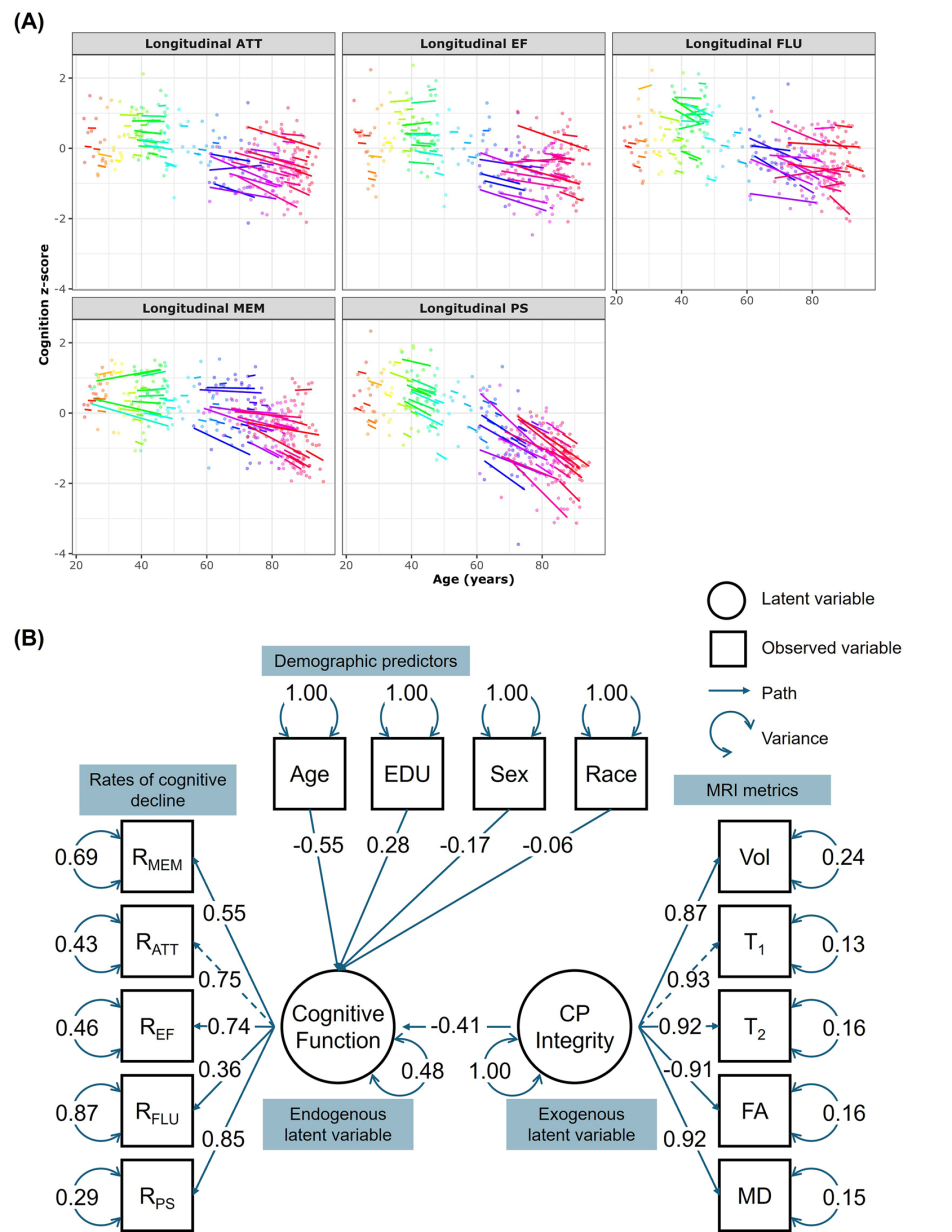
Structural equation modeling (SEM) was applied to investigate the relationship between combined MRI metrics (CPI) and combined rates of cognitive decline (CogFun). **Panel A** displays the estimated rates of cognitive decline for each subject, derived from linear mixed-effects models: ${\rm{Cognitio}}{{\rm{n}}_{{\rm{ij}}}}\,{\rm{ = }}\,{{\rm{\beta }}_{\rm{0}}}\,{\rm{ + }}\,{{\rm{\beta }}_{{\rm{Time}}}}\,{\rm{ \times }}\,{\rm{Tim}}{{\rm{e}}_{{\rm{ij}}}}\,{\rm{ + }}\,{{\rm{b}}_{{\rm{Time,i}}}}\,{\rm{ \times }}\,{\rm{Tim}}{{\rm{e}}_{{\rm{ij}}}}\,{\rm{ + }}\,{ \in _{{\rm{ij}}}}\,{\rm{ + }}\,{{\rm{b}}_{\rm{i}}}$. The rate of cognitive decline is defined as the sum of the fixed effect (β_Time_) and the subject-specific random effect (b_Time,i_). Each subject is color-coded and has a fitted decline line if more than two longitudinal measurements exist. **Panel B** visualizes the SEM results. The measurement model for CP integrity, CPI (exogenous latent variable), is defined as: ${\rm{CPI}}\,{\rm{ = }}\,{{\rm{\lambda }}_{{{\rm{T}}_{\rm{1}}}}}\,{\rm{ \times }}\,{{\rm{T}}_{\rm{1}}}\,{\rm{ + }}\,{{\rm{\lambda }}_{{{\rm{T}}_{\rm{2}}}}}\,{\rm{ \times }}\,{{\rm{T}}_{\rm{2}}}\,{\rm{ + }}\,{{\rm{\lambda }}_{{\rm{FA}}}}\,{\rm{ \times }}\,{\rm{FA}}\,{\rm{ + }}\,{{\rm{\lambda }}_{{\rm{MD}}}}\,{\rm{ \times }}\,{\rm{MD}}\,{\rm{ + }}\,{{\rm{\lambda }}_{{\rm{Vol}}}}\,{\rm{ \times }}\,{\rm{Vol}}\,{\rm{ + }}\, \in$, where λ represents factor loadings, and ϵ represents residual variance. Similarly, the measurement model for combined change rates of cognitive-function, Cog-fun (endogenous latent variable), is defined as: ${\rm{CogFun}}\,{\rm{ = }}\,{{\rm{\lambda }}_{{\rm{MEM}}}}\,{\rm{ \times }}\,{{\rm{R}}_{{\rm{MEM}}}}\,{\rm{ + }}\,{{\rm{\lambda }}_{{\rm{ATT}}}}\,{\rm{ \times }}\,{{\rm{R}}_{{\rm{ATT}}}}\,{\rm{ + }}\,{{\rm{\lambda }}_{{\rm{EF}}}}\,{\rm{ \times }}\,{{\rm{R}}_{{\rm{EF}}}}\,{\rm{ + }}\,{{\rm{\lambda }}_{{\rm{FLU}}}}\,{\rm{ \times }}\,{{\rm{R}}_{{\rm{FLU}}}}\,{\rm{ + }}\,{{\rm{\lambda }}_{{\rm{PS}}}}\,{\rm{ \times }}\,{{\rm{R}}_{{\rm{PS}}}}\,{\rm{ + }}\, \in$, where R_MEM_, R_ATT_, R_EF_, R_FLU_, R_PS_ are rates of decline in memory, attention, executive function, fluency, and processing speed, respectively. The structural regression model is: ${\rm{CogFun}}\,{\rm{ = }}\,{{\rm{\beta }}_{{\rm{CPI}}}}\,{\rm{ \times }}\,{\rm{CPI}}\,{\rm{ + }}\,{{\rm{\beta }}_{{\rm{Age}}}}\,{\rm{ \times }}\,{\rm{Age}}\,{\rm{ + }}\,{{\rm{\beta }}_{{\rm{Sex}}}}\,{\rm{ \times }}\,\,{\rm{Sex}}\,{\rm{ + }}\,{{\rm{\beta }}_{{\rm{EDU}}}}\,{\rm{ \times }}\,{\rm{EDU}}\,{\rm{ + }}\,{{\rm{\beta }}_{{\rm{Race}}}}\,{\rm{ \times }}\,{\rm{Race}}\,{\rm{ + }}\, \in$. The results are presented in the standardized form, allowing the parameter estimates to be interpreted as standardized effects. The measurement model for CPI indicates that all MRI metrics have significant standardized loadings, with CP volume contributing less to the latent variable because it reflects macrostructure, while other MRI metrics probe microstructure. Additionally, FA shows a negative loading because, unlike other MRI metrics, lower FA values indicate greater microstructural damage. In the measurement model for CogFun, all rates of cognitive domain changes have significant loadings, with fluency contributing the least. The structural model reveals that CPI has a significant negative effect on CogFun (β = −0.41, *p* < 0.001), suggesting that higher CPI predicts faster cognitive decline. This effect is comparable to that of age (β = −0.55, *p* < 0.001), indicating a strong association. Education (β = 0.28, *p* = 0.001) has a protective effect, while the effects of sex (β = −0.17, *p* = 0.045) and race (β = −0.06, *p* = 0.456) are weaker, with race being nonsignificant

Finally, Fig. 3 shows the results of the SEM analysis investigating the relationship between combined MRI metrics (CPI) and combined change rates of cognitive function (CogFun) (Eqs. 3–5). As expected, derived rates of cognitive change exhibit decreased trends as a function of age, especially at older ages (Fig. 3A). Our analysis reveals that the measurement model for CP damage indicates significant standardized loadings for all MRI metrics. Interestingly, CP volume, a measure of macrostructural integrity, contributes the least to the latent variable as compared to all other MRI metrics, which are measures of microstructural alterations. Of note, FA exhibits a negative loading, as lower FA values reflect worse microstructural integrity. In the measurement model for cognitive changes, all rates of cognitive domain changes display significant loadings, with fluency contributing the least. The structural model reveals that CPI is negatively associated with CogFun (β = −0.41, *p* < 0.001), indicating that worse overall CP structural integrity predicts faster cognitive decline. This effect is close to that of age (β = −0.55, *p* < 0.001), highlighting a strong association. Furthermore, education exhibits a protective effect (β = 0.28, *p* = 0.001), whereas the effects of sex (β = −0.17, *p* = 0.045) and race (β = −0.06, *p* = 0.456) are weaker, with the latter being nonsignificant.

## Discussion

The present study revealed significant associations between CP integrity, as measured by MRI metrics, and cognitive function across various domains. Notably, our results showed that CP volume, T_1_, T_2_, and MD values were negatively correlated with cognitive performance, whereas FA values exhibited positive associations, indicating that compromised CP structural integrity is linked to poorer cognitive function. These findings were statistically significant for PS and FLU, suggesting that CP integrity plays a critical role in maintaining optimal cognitive performance. In the SEM analysis, CP volume exhibited the lowest loading on the CPI, suggesting that macrostructural change contributed less to overall CP integrity than microstructural measures such as FA and MD. This pattern is consistent with a temporal cascade in which microstructural degeneration typically precedes measurable macrostructural atrophy, making microstructural indices more sensitive to early or subtle integrity changes in cognitively normal individuals. Volume differences may become more influential at later stages of neurodegeneration, whereas microstructural markers capture earlier cellular or vascular alterations. Furthermore, our analysis revealed that the associations between CP integrity and cognitive function were influenced by demographic factors, such as age, sex, education, and race, highlighting the importance of considering individual differences and sociological disparities related to demographic factors when examining the neural correlates of cognitive function. The observed relationships between CP integrity and cognitive decline also underscore the potential utility of MRI metrics as biomarkers for monitoring cognitive changes and detecting early signs of neurodegenerative diseases. Overall, our findings contribute to a deeper understanding of the complex interplay between brain structure and cognitive function and have significant implications for the development of novel diagnostic and therapeutic strategies aimed at promoting cognitive health and mitigating cognitive decline.

Our study focused on cognitively normal adults to examine whether microstructural alterations of the CP can be detected before the onset of clinically significant cognitive impairment. This design allowed us to identify subtle CP changes that associate with differential longitudinal cognitive decline. By limiting our sample to cognitively normal individuals, we minimized confounds related to widespread pathology and atrophy present in MCI or AD and demonstrated that CP integrity is relevant even in the absence of overt disease. Moreover, the GESTALT and BLSA cohorts were specifically designed to study normative aging and included very few participants with MCI or AD, precluding adequately powered subgroup analyses. Taken together, our findings highlight that CP microstructural alterations are detectable in preclinical stages of aging and are meaningfully associated with cognitive function, supporting their potential role as early biomarkers of cognitive vulnerability.

Existing evidence shows the sensitivity of microstructural changes to cognitive decline, which precedes macrostructural changes by decades [41, 42]. By leveraging advanced MRI techniques, including diffusion and relaxometry, we demonstrate that microstructural alterations in the CP are more sensitive indicators of CP dysfunction and cognitive decline than macrostructural measurement. Previous research highlights the association between CP volume and cognitive impairment in various conditions. However, our study addresses a significant gap in literature by investigating the relationship between CP microstructural integrity and cognitive decline in normative aging, using qMRI techniques. While previous studies have relied on structural MRI to examine the relationship between CP volume and cognitive performance [9, 10, 14, 25, 43–45], our study provides novel insights into the microstructural changes underlying CP dysfunction and cognitive decline.

The mechanisms underlying the association between CP degeneration and cognitive decline are complex and multifaceted. Previous studies suggest that CP dysfunction may contribute to cognitive decline through various pathways, including disrupted CSF dynamics [1, 46, 47], neuroinflammation [48–50], and impaired glymphatic function [21, 51, 52]. The age-related increase in CP volume may lead to an inflammatory response to the blood/CP-CSF barrier as well as in the brain itself. Furthermore, the CPs role in regulating the entry of substances into the CNS and facilitating the removal of waste products from the brain may be compromised with aging [46, 47, 53, 54], leading to the accumulation of toxic substances and exacerbating cognitive decline. Additionally, the association between increased CP volume and impaired glymphatic function may also contribute to cognitive decline [21, 51, 52], as the glymphatic system plays a critical role in clearing metabolites from the brain. Further studies are needed to elucidate the underlying mechanisms and to determine the causal relationships between CP degeneration, neuroinflammation, and cognitive decline.

We note that in the present study, MRI scans were performed at the end of the cognitive assessment period. While this design allows us to relate CP structural integrity to the trajectory of cognitive changes, it limits our ability to establish causality. Specifically, the observed CP alterations could represent early contributors to cognitive decline, but they could also reflect adaptive or reactive changes to ongoing parenchymal alterations. Accordingly, while our findings demonstrate a strong association between CP microstructural integrity and cognitive decline, they do not definitively establish whether CP changes precede or follow cognitive deterioration. Future studies incorporating longitudinal MRI assessments at multiple time points are needed to determine whether CP structural changes can serve as early predictive biomarkers for cognitive decline in normative aging.

Although our study provides novel clinical insights into the relationship between CP structural integrity and cognitive decline in normative aging, some limitations should be acknowledged. First, our study employed a single time-point MRI assessment, which precludes the examination of longitudinal changes in CP structural integrity. Future studies incorporating longitudinal MRI assessments are necessary to verify the temporal relationships between CP structural changes and cognitive decline. Second, our study relied on indirect MRI markers of CP microstructural integrity, which cannot fully capture the complex cellular and molecular changes occurring within the CP. The development of more sensitive and specific biomarkers is needed to further elucidate the mechanisms underlying CP dysfunction and cognitive decline. Third, the diffusion MRI protocol used a moderate b-value (700 s/mm^2^) consistent with the standardized BLSA/GESTALT acquisition protocol. While this setting ensured high signal-to-noise ratio and reliable tensor estimation, particularly for the small and CSF-adjacent choroid plexus, it may have limited sensitivity to more subtle microstructural features that could be captured with higher b-values. Future studies employing multi-shell or higher-b diffusion imaging may provide a more detailed characterization of CP microstructure. Finally, our study did not control for potential confounding variables, such as sleep quality, physical activity, and social engagement, which may influence the control between CP microstructural integrity and cognitive decline. Future studies should strive to incorporate these variables to provide a more comprehensive understanding of the complex interplay between CP function, lifestyle factors, and cognitive health.

## Conclusions

Our findings demonstrate that compromised CP structural integrity is significantly associated with cognitive decline in aging among cognitively unimpaired individuals. This underscores the importance of CP health in maintaining cognitive function and suggests that advanced CP imaging biomarkers could aid in early detection and intervention strategies for cognitive decline.

## Electronic supplementary material

Below is the link to the electronic supplementary material.

Supplementary Material 1

Supplementary Material 2

Supplementary Material 3

## Data Availability

The datasets used and/or analyzed during the current study are available from the corresponding author on reasonable request.
