# Supplementary material for: Age-related differences in choroid plexus structural integrity are associated with changes in cognition
Source: Fluids Barriers CNS. 2025 Dec 18;23:14. doi: 10.1186/s12987-025-00749-3 (PMC12829252; doi:10.1186/s12987-025-00749-3)
Supplement: Supplementary file 3 — Supplementary Material 3 [file 12987_2025_749_MOESM3_ESM.docx]

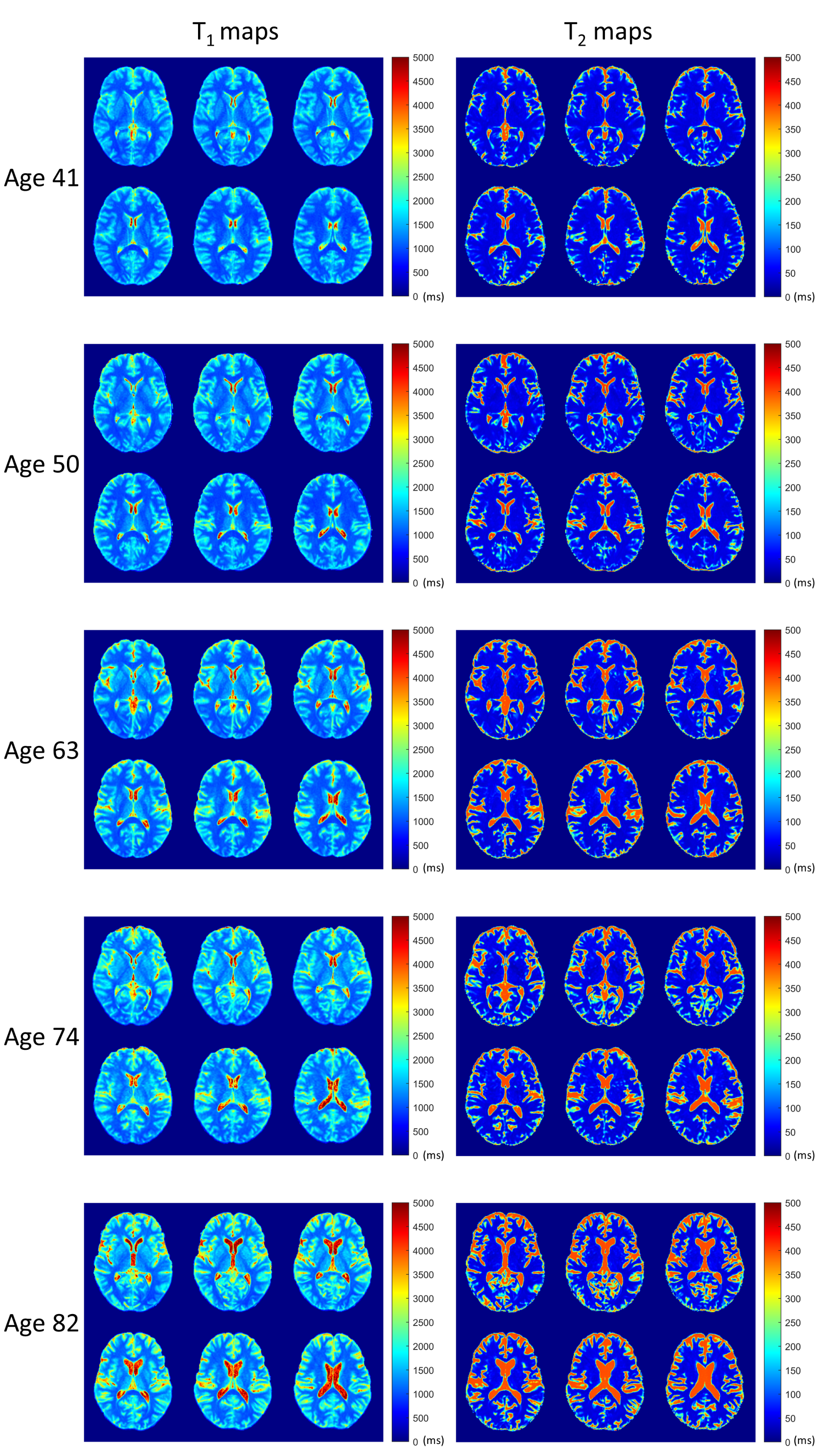


Figure S1. With advancing age, the choroid plexus exhibits higher values in T_1_ and T_2_, converging toward values in adjacent ventricular regions. This pattern is consistent with an age-related loss of microstructural integrity within the CP.


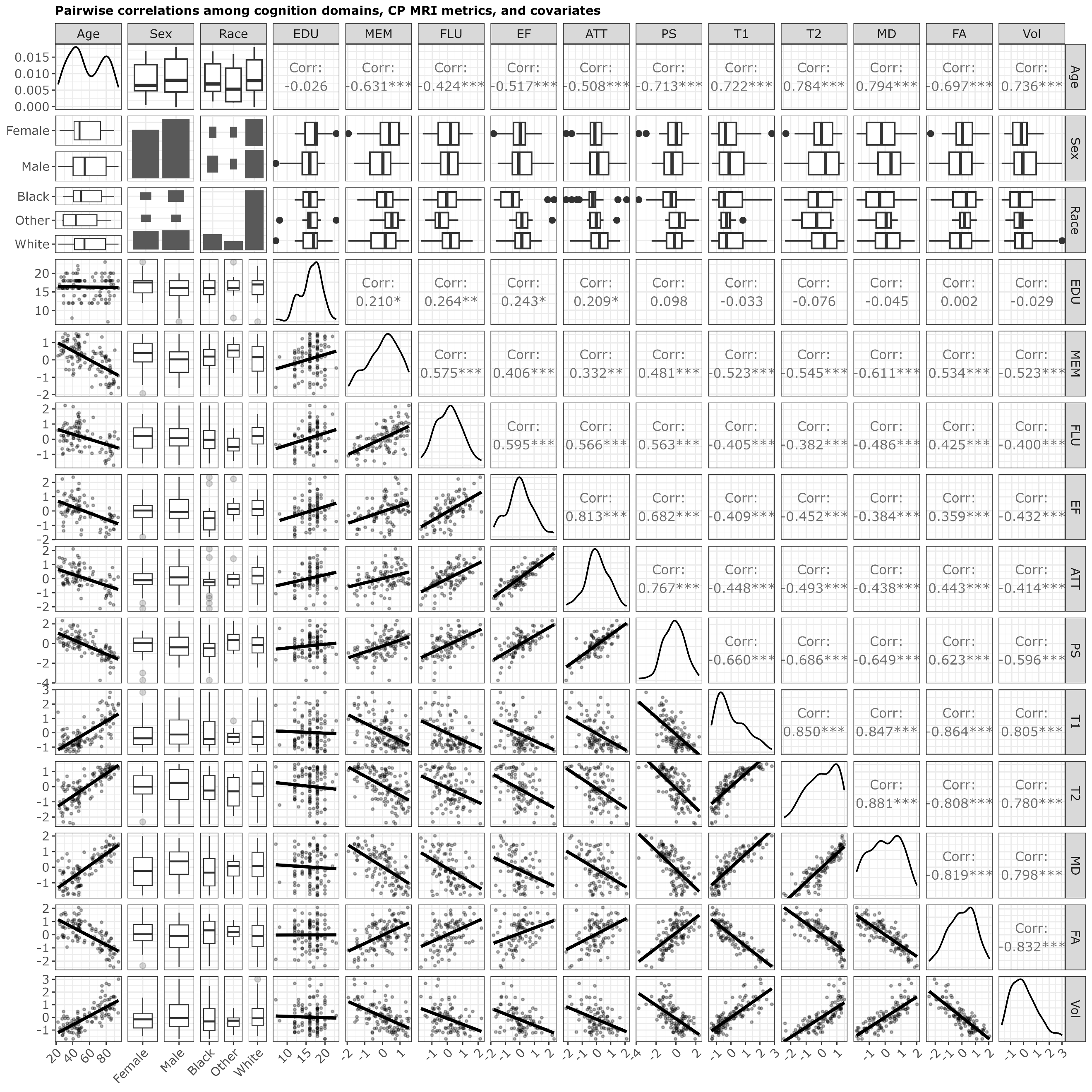


Figure S2. Pairwise linear correlations among Cognition, CP MRI metrics and covariates. T_1_, T_2_, MD and CP volume negatively correlate with cognition scores, while FA values positively correlate with cognition scores. This observation promoted the full linear regression analyses in the main manuscript.


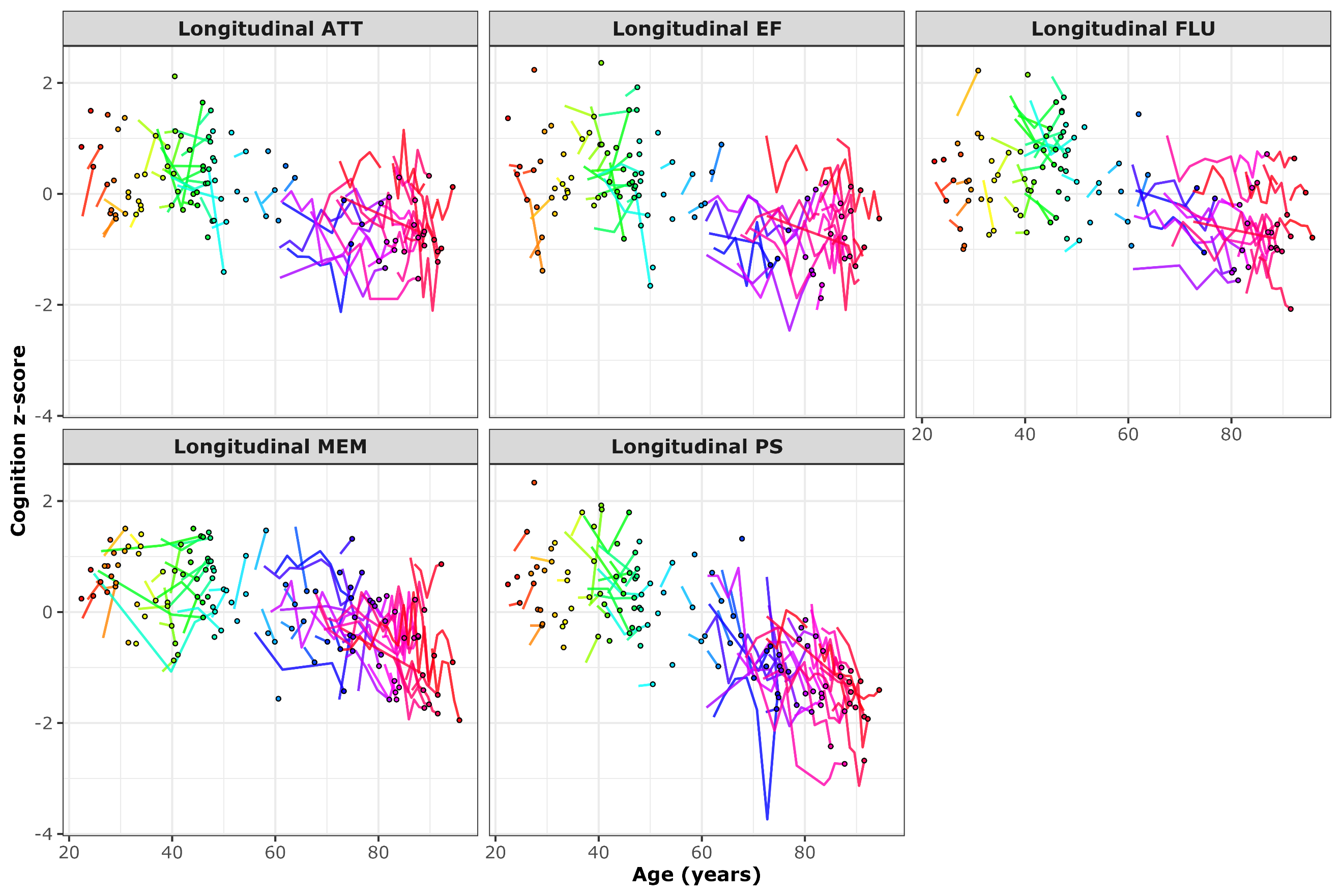


Figure S3. Longitudinal decline curve for cognitions. The age of MRI scans is highlighted by the points. A general trend of cognitive decline is visible for those with longitudinal assessments.


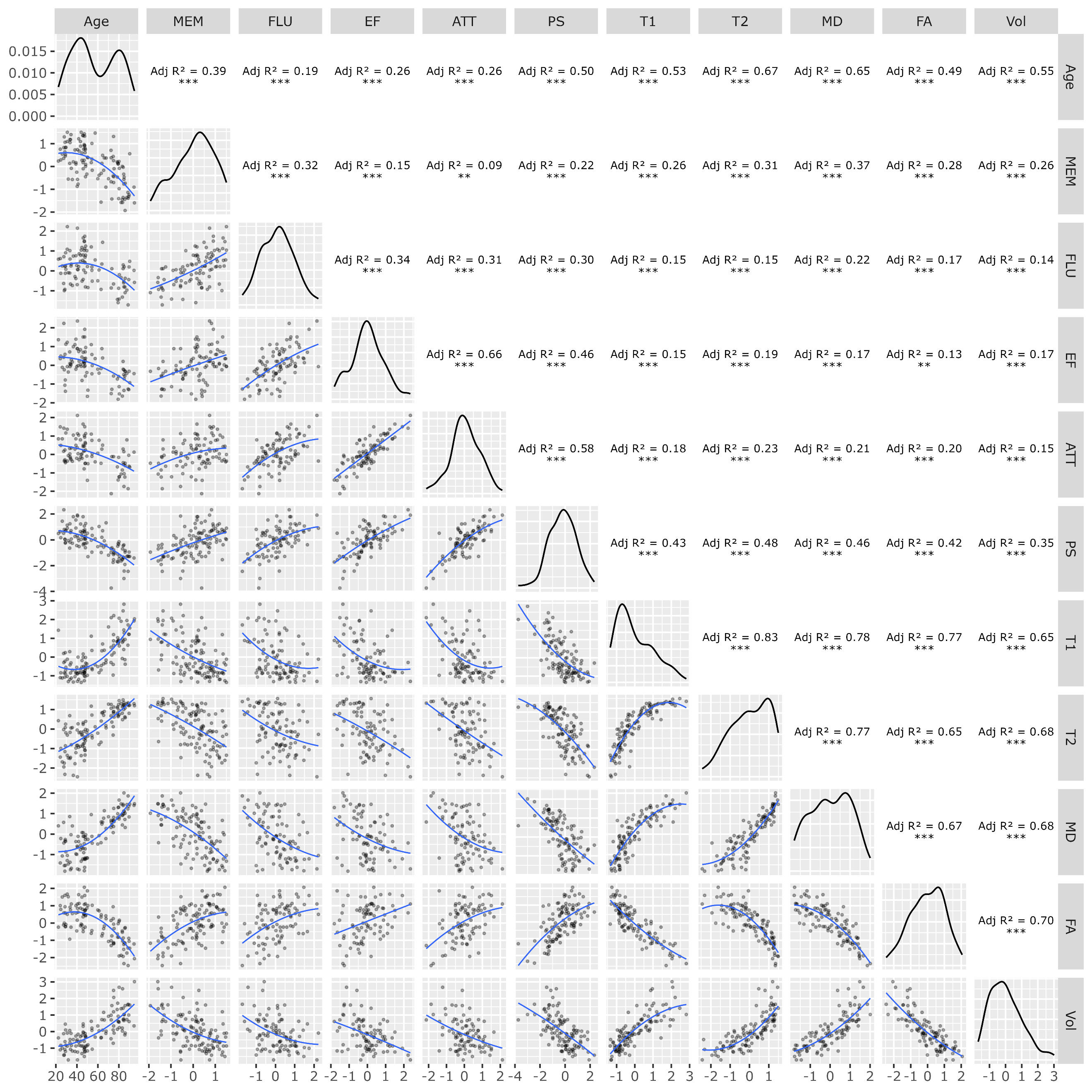


Figure S4. Pairwise quadratic correlations among Cognition, CP MRI metrics and covariates. A small degree of quadratic correlation is observed for most pairwise relationships. However, a larger cohort is still needed to explore this nonlinear relationship in detail for future studies.
